# Effects of 4 multitargeted receptor tyrosine kinase inhibitors on regional hemodynamics in conscious, freely moving rats

**DOI:** 10.1096/fj.201600749R

**Published:** 2016-12-16

**Authors:** Joanne J. Carter, Laurice V. Fretwell, Jeanette Woolard

**Affiliations:** * Cell Signalling Research Group, School of Life Sciences, Medical School, The University of Nottingham, Queen’s Medical Centre, Nottingham, United Kingdom; and; † Faculty of Health and Life Sciences, De Montfort University, Leicester, United Kingdom

**Keywords:** cancer therapy, hypertension, RTKI, VEGF, regional hemodynamics

## Abstract

VEGF inhibitors, including receptor tyrosine kinase inhibitors, are used as adjunct therapies in a number of cancer treatments. An emerging issue with these drugs is that most cause hypertension. To gain insight into the physiological mechanisms involved, we evaluated their regional hemodynamic effects in conscious rats. Male Sprague Dawley rats (350–450 g) were chronically implanted with pulsed Doppler flow probes (renal and mesenteric arteries, and the descending abdominal aorta) and catheters (jugular vein, peritoneal cavity, and distal abdominal aorta). Regional hemodynamics were measured over 4 d, before and after daily administration of cediranib (3 and 6 mg/kg, 3 and 6 mg/kg/h for 1 h, i.v.), sorafenib (10 and 20 mg/kg, 10 and 20 mg kg/h for 1 h, i.v.), pazopanib (30 and100 mg/kg, i.p.), or vandetanib (12.5 and 25 mg/kg, i.p.). All drugs evoked significant increases (*P* < 0.05; *n* = 7–8) in mean arterial pressure, which were generally accompanied by significant mesenteric and hindquarters, but not renal, vasoconstrictions. The hypertensive effects of cediranib were unaffected by losartan (10 mg/kg/h), bosentan (20 mg/kg/h), or a combination of phentolamine and propranolol (each 1 mg/kg/h), suggesting a need for new strategies to overcome them.—Carter, J. J., Fretwell, L. V., Woolard, J. Effects of 4 multitargeted receptor tyrosine kinase inhibitors on regional hemodynamics in conscious, freely moving rats.

Vascular endothelial growth factor (VEGF) is an important mediator of cell survival, proliferation, and angiogenesis (1–4). As a consequence, VEGF and its receptors have become a major focus of cancer research over the last decade (4). Inhibitors of VEGF activity, including monoclonal antibodies and receptor tyrosine kinase inhibitors (RTKIs), have been developed to interfere with tumor angiogenesis and many are in the clinic as adjunct therapies to improve cancer prognosis (5–7).

Bevacizumab (Avastin; Genentech, San Francisco, CA, USA), a humanized anti-VEGF antibody, is currently used in the treatment of metastatic carcinoma of colon or rectum, lung (non-small cell), and kidney (5). The RTKIs sunitinib and sorafenib have been licensed for use in advanced renal cell carcinoma and gastrointestinal stromal tumors (8), while cediranib is undergoing preclinical testing for ovarian cancer (9), and vandetanib is being used to treat medullary thyroid cancer (10). These inhibitors were designed to be less specific, multitargeted agents compared to monoclonal antibodies, and although these approaches were expected to cause minimal adverse effects, this does not appear to be the case (8, 11–13).

An emerging issue with anti-VEGF therapies is the development of significant hypertension, leading to left ventricular dysfunction and heart failure after longer-term exposure over several months (11–13). Approximately 20 to 30% of patients treated with the monoclonal antibody bevacizumab (5) and 15 to 60% of patients treated with small-molecule multitargeted RTKIs experienced treatment-associated hypertension (13–17). Moreover, the development and/or escalation of preexisting hypertension in these patients has been linked to multiple severe complications, including venous or arterial thromboembolism, acute heart failure, and intracerebral hemorrhage (18–20). Thus, an elevation in blood pressure (BP) often leads to treatment cessation or possible life-threatening adverse events (13).

Some attempts have been made to recapitulate these hypertensive effects in small animal models in order to provide insight into the potential mechanisms involved (21–23). In telemetered rats, it was demonstrated that 8 d of treatment with the RTKI, sunitinib-induced an approximate 30 mmHg rise in BP, which was associated with an elevation in circulating endothelin-1 and creatinine levels (21). Furthermore, functional studies in coronary microvessels isolated from these treated animals demonstrated reduced responsiveness to bradykinin, angiotensin II, and sodium nitroprusside; this loss of functionality was not seen in preparations where sunitinib treatment was withdrawn 11 d before experimentation (21). Additional animal studies have suggested that sunitinib has no direct effects on cardiac structure and/or function (22). More recently, the effects of sunitinib and 2 other RTKIs, cediranib and sorafenib, on BP were evaluated in rats instrumented with radiotelemetric devices (23). All 3 VEGF signaling inhibitors showed hypertensive effects over a range of doses, where a change in diastolic pressure was used as an index of vasoconstriction. This ability to demonstrate hypertensive effects in nonclinical *in vivo* models suggests that further whole animal approaches should be useful in unraveling the mechanisms underlying the development of RTKI-induced clinical hypertension (23).

In the context of animal studies, to our knowledge, no one has shown whether the onset of hypertension after RTKI administration is a cardiac or vascular event because all previous approaches have been confined to the use of implanted radiotelemetric devices, which are limited to measurements of BP and heart rate (HR). The aims of the current study were therefore to determine the following in conscious rats: how early (0–4 d) hypertensive effects could be observed with different RTKIs; whether these effects were associated with vasoconstriction; and if this vasoconstriction was regionally selective. The model chosen allowed vascular conductance (VC) to be measured simultaneously in 3 different vascular beds using Doppler flow probes sutured around the renal and mesenteric arteries and the descending aorta (24–26). The 4 RTKIs chosen have been previously shown to inhibit VEGF receptor 2 (VEGFR2)-mediated reporter gene responses with a rank order of potency of cediranib > pazopanib > sorafenib > vandetanib (27). Because we were able to show regionally selective vasoconstrictor effects that were particularly marked with cediranib, we then investigated whether the cardiovascular effects of cediranib could be prevented by antagonism of angiotensin AT_1_ receptors (AT_1_Rs), endothelin-1 receptors, or adrenoceptors.

## MATERIALS AND METHODS

### Animals and surgery

Adult male Sprague Dawley rats (Charles River Laboratories, Wilmington, MA, USA) weighing 350 to 450 g were housed in groups in a temperature-controlled (21–23°C) environment with a 12-h light–dark cycle (lights on at 6:00 am) and free access to food (18% Protein Rodent Diet; Teklad Global, Bicester, United Kingdom) and water for at least 7 d after arrival from the supplier before any surgical intervention.

Surgery was performed in 2 stages under general anesthesia (fentanyl and medetomidine, 300 μg/kg each, i.p., supplemented as required), with reversal of anesthesia and postoperative analgesia provided by atipamezole (1 mg/kg, s.c.) and buprenorphine (0.02 mg/kg, s.c.). At the first surgical stage, miniature pulsed Doppler flow probes were sutured around the renal and mesenteric arteries and the descending abdominal aorta to monitor hemodynamics. The wires from the probes were taped and sutured at the nape of the neck, and the animals were returned to the holding room. At the second surgical stage, which took place at least 10 d after the surgery for probe implantation, and after a satisfactory inspection from the Named Veterinary Surgeon, catheters were implanted in the distal abdominal aorta *via* the caudal artery (for arterial BP monitoring and the derivation of HR) and in the right jugular vein (for drug administration). Three separate intravenous catheters were placed in the jugular vein to enable concurrent administration of different substances. In some experiments, intraperitoneal catheters were inserted through the abdominal wall. At this stage, the wires from the probes were soldered into a miniature plug (Microtech, Boothwyn, PA, USA), which was mounted onto a custom-designed harness worn by the rat. The catheters emerged from the same point as the probe wires and were fed through a protective spring secured to the harness and attached to a counterbalanced pivot system. The arterial catheter was connected to a fluid-filled swivel for overnight infusion of heparinized (15 U/ml) saline to maintain potency.

Experiments began 24 h after surgery for catheter implantation, with animals fully conscious and unrestrained in home cages, and with free access to food and water. All procedures were carried out with approval of the University of Nottingham Animal Welfare Ethical Review Board under Home Office Project and Personal License Authority.

### Cardiovascular recordings

Cardiovascular variables were recorded using a customized, computer-based system (IdeeQ; Maastricht Instruments, Maastricht, The Netherlands) that connected a transducer amplifier (13-4615-50; Gould, Cleveland, OH, USA), a Doppler flowmeter (Crystal Biotech, Holliston, MA, USA), and a VF-1 mainframe (pulse repetition frequency 125 kHz) fitted with high-velocity (HVPD-20) modules. Raw data were sampled by IdeeQ every 2 ms, averaged, and stored to disc every cardiac cycle. Changes in renal VC (RVC), mesenteric VC (MVC), and hindquarter VC (HVC), in the renal, mesenteric, and hindquarter vascular beds, respectively, were calculated from the changes in mean arterial pressure (MAP) and Doppler shift.

### Experimental protocol

Experiments were run in 4 series; within each series was a contemporaneous control. Experiments were generally run with treatment groups of 6 to 8 rats.

#### Series 1

Six groups of rats were used. On d 1 of the experiment, after a period of baseline recording, rats were provided cediranib (3 or 6 mg/kg) or sorafenib (10 or 20 mg/kg) as an intravenous bolus (0.1 ml provided over 5 s) followed by a 1-h intravenous infusion (0.4 ml/h) at the same dose. Contemporaneous controls were provided vehicle (5% propylene glycol, 2% Tween 80 in sterile saline). Recordings were continued for a further 3 h after completion of the intravenous infusion period. The same treatment regimen was followed on d 2–4 after a period of baseline recording on each day.

#### Series 2

Six groups of animals were used. Rats were provided vandetanib (12.5 or 25 mg/kg) or pazopanib (30 or 100 mg/kg) as an intraperitoneal bolus (0.1 ml provided over 5 s). Solubility issues with both drugs prevented utilization of the intravenous protocol described above for cediranib and sorafenib. Instead, both drugs were administered as single intraperitoneal bolus injections. Contemporaneous controls were provided vehicle (5% propylene glycol, 2% Tween 80 in sterile saline, i.p.). Cardiovascular recordings were continued for a further 4 h after administration of the compounds. The same treatment regimen was repeated on d 2–4 after a period of baseline recording on each day.

#### Series 3

Two groups of animals were used. Rats were provided the AT_1_R antagonist losartan (10 mg/kg) as an intravenous bolus (0.1 ml) followed by an intravenous infusion (10 mg/kg/h; 0.4 ml/h) over the entire monitoring period (28). Control animals were provided vehicle (sterile saline). One hour after commencement of the losartan or vehicle infusion, cediranib (3 mg/kg, 3 mg/kg/h for 1 h) was provided to all animals. Recordings were then continued for a further 3 h. The same treatment regimen was repeated on d 2, with a final recording being taken on d 3.

#### Series 4

Three groups of animals were used. Rats were provided the endothelin-1 receptor antagonist bosentan (20 mg/kg; 20 mg/kg/h infusion) (24) or a combination of the α- and β-adrenoceptor antagonists phentolamine (1 mg/kg; 1 mg/kg/h infusion) and propranolol (1 mg/kg; 0.5 mg/kg/h infusion) (29). Control animals were provided the appropriate vehicle (5% propylene glycol, 2% Tween 80 in sterile saline for bosentan, and saline for phentolamine/propranolol). Vehicle, bosentan, or phentolamine/propranolol infusions were continued for 5 h. One hour after commencement of the drug or vehicle infusion, cediranib (3 mg/kg, 3 mg/kg/h) was provided to all animals. The same treatment regimen was repeated for each rat on d 2, with a final baseline recording being taken on d 3. In the bosentan-treated group, endothelin (1 μM as a 0.1 ml bolus in 1% bovine serum albumin/saline) was routinely administered at the end of d 2 in all of our experiments to verify that the cardiovascular effects had been significantly attenuated by the infusion of bosentan (24).

### Data analysis

Data were analyzed offline using IdeeQ software. For all experiments, time-averaged data are shown as changes from baseline [HR (beats/min); MAP (mmHg); VC (%)]. Statistical comparisons between groups of animals were performed on the integrated changes over specified time periods. Because the data were not all normally distributed, a nonparametric, repeated-measures analysis of variance (Friedman’s test) (30) was used for within-group comparisons and Mann-Whitney *U* test for between-group comparisons, as appropriate. Vascular conductances were calculated from the MAP and Doppler shift (flow) data. A value of *P* < 0.05 was considered significant. Each animal represented 1 experimental unit.

## RESULTS

Baseline cardiovascular variables before the administration of RTKIs and corresponding vehicles are shown in **Table 1**.

**TABLE 1.** Baseline cardiovascular variables before administration of vehicle or RTKI

Series	*n*	HR (beats/min)	MAP (mmHg)	RDS (kHz)	RVC (U)	MDS (kHz)	MVC (U)	HDS (kHz)	HVC (U)
Series 1									
Vehicle 1	7	362 ± 16	106 ± 4	8.8 ± 0.5	83 ± 4	9.8 ± 1.4	93 ± 14	5.4 ± 0.4	51 ± 3
Cediranib, low dose	8	359 ± 8	106 ± 4	8.6 ± 0.9	82 ± 8	9.3 ± 0.6	90 ± 8	6.0 ± 0.5	57 ± 4
Sorafenib, low dose	8	336 ± 9	103 ± 4	8.9 ± 0.9	87 ± 9	9.2 ± 1.2	89 ± 11	5.9 ± 0.4	58 ± 6
Vehicle 2	8	374 ± 12	107 ± 3	7.8 ± 1.3	72 ± 12	9.2 ± 1.5	86 ± 13	5.4 ± 0.6	51 ± 6
Cediranib, high dose	8	357 ± 8	105 ± 3	8.3 ± 0.9	79 ± 7	8.4 ± 1.4	82 ± 15	5.4 ± 0.3	51 ± 3
Sorafenib, high dose	7	347 ± 4	102 ± 5	9.3 ± 0.9	90 ± 5	7.5 ± 1.0	74 ± 9	5.6 ± 0.4	55 ± 5
Series 2									
Vehicle 3	8	354 ± 5	104 ± 3	8.9 ± 1.4	86 ± 14	11.4 ± 1.4	110 ± 15	5.8 ± 0.4	57 ± 5
Vandetanib, low dose	8	368 ± 4	98 ± 2	8.8 ± 0.9	90 ± 9	10.0 ± 1.3	102 ± 12	6.2 ± 0.5	64 ± 7
Vandetanib, high dose	8	357 ± 11	103 ± 3	8.5 ± 1.0	81 ± 7	12.5 ± 1.2	122 ± 12	6.3 ± 0.7	62 ± 8
Vehicle 4	8	356 ± 15	105 ± 3	10.1 ± 1.6	96 ± 14	9.9 ± 1.1	95 ± 12	6.0 ± 0.3	58 ± 3
Pazopanib, low dose	8	360 ± 6	97 ± 1	9.2 ± 0.5	95 ± 5	11.4 ± 1.2	118 ± 13	5.5 ± 0.4	57 ± 4
Pazopanib, high dose	8	351 ± 5	103 ± 3	8.1 ± 0.6	78 ± 4	11.2 ± 0.4	109 ± 6	5.3 ± 0.4	53 ± 5
Series 3									
Vehicle 5 + cediranib	6	372 ± 12	105 ± 4	9.1 ± 0.7	88 ± 6	7.2 ± 0.7	69 ± 6	4.5 ± 0.3	44 ± 4
Losartan + cediranib	8	382 ± 15	99 ± 2	9.5 ± 0.6	96 ± 6	10.4 ± 0.8	104 ± 7	5.3 ± 0.3	54 ± 4
Series 4 baseline values:									
Vehicle 6 + cediranib	6	347 ± 10	107 ± 8	11.2 ± 3.0	102 ± 20	9.4 ± 1.2	90 ± 17	5.5 ± 0.4	51 ± 3
Bosentan + cediranib	4	365 ± 8	99 ± 3	8.1 ± 2.4	80 ± 23	10.4 ± 1.4	107 ± 16	5.8 ± 0.9	58 ± 8
Phentolamine/propranolol + cediranib	7	351 ± 13	104 ± 3	7.4 ± 1.2	71 ± 12	11.6 ± 0.9	111 ± 9	6.3 ± 0.4	60 ± 4

Data show baseline cardiovascular variables. Measurements (means ± sem) were made immediately before administration of drugs or vehicle according to 4 experimental series described in Materials and Methods. *n*, number of animals in each experimental group. Units for VC are kHz ⋅ mmHg · 10^−3^. RDS, renal Doppler shift; MDS, mesenteric Doppler shift; HDS, hindquarters Doppler shift.

### Series 1: Effects of cediranib and sorafenib

**Figure 1.**
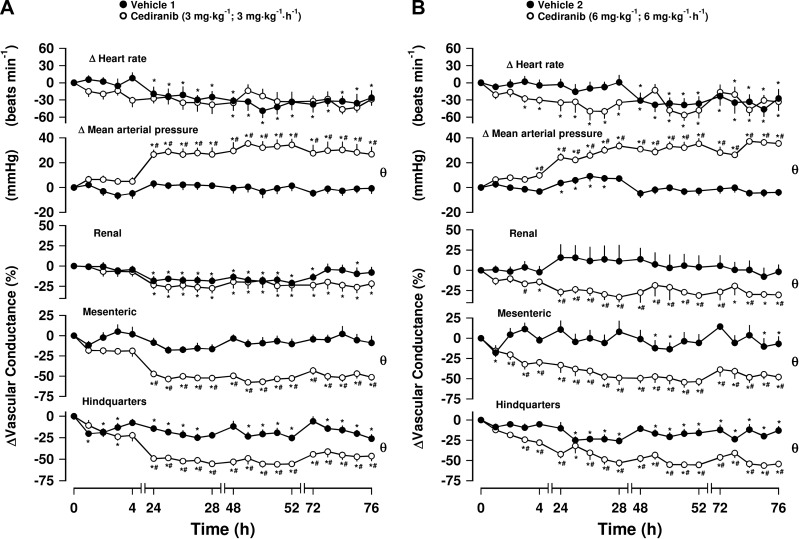
Cardiovascular responses to cediranib in conscious, freely moving rats. Rats were dosed with either 3 mg/kg, i.v. (initial bolus followed by 3 mg/kg/h, i.v. infusion for 1 h; *n* = 8) (*A*) or 6 mg/kg, i.v. (initial bolus followed by 6 mg/kg/h, i.v. infusion for 1 h; *n* = 8) cediranib (*B*). Vehicle controls (*n* = 7, *A*; *n* = 8, *B*) were administered 5% propylene glycol, 2% Tween 80 in sterile saline) as described in Materials and Methods. Data points are means; vertical bars represent sem. θ, significant difference between-group comparison (*P* < 0.05; Mann-Whitney *U* test, 0–76 h) based on integrated area under or above curve analysis. Where both above tests were positive, Mann-Whitney *U* test was conducted between treated and vehicle control groups at each timepoint. *P < 0.05 *vs*. baseline (Friedman’;s test); ^#^*P* < 0.05 *vs*. vehicle control (Mann-Whitney *U* test).

Administration of vehicle caused no consistent cardiovascular effects in either group, although there was some reduction in HR and HVC across the 4-d recording period (**Fig. 1**, Friedman’s test). Cediranib at both 3 and 6 mg/kg produced a sustained elevation of MAP over the 4-d experimental period (Fig. 1, Friedman’s test); however, neither dose of cediranib produced a consistent change in HR. At both doses, the pressor effect was accompanied by significant vasoconstrictions in the mesenteric and hindquarters vascular beds (Fig. 1). At the lower dose of cediranib (3 mg/kg; 3 mg/kg/h), no significant change was seen in RVC (Fig. 1*A*), but at the higher dose (6 mg/kg; 6 mg/kg/h), there was a significant renal vasoconstriction (Fig. 1*B*). These treatment-induced pressor and vascular effects were significantly different from the vehicle group over the 4-d experimental period (Mann-Whitney *U* test, integrated area under curve, 0–76 h). In cases in which both Friedman and Mann-Whitney tests were positive, we conducted an additional Mann-Whitney test between treated and vehicle groups at each time point to determine the time of onset of the cardiovascular effects. For almost all points of analysis, there was a significant difference between the 2 groups after 24 h (*P* < 0.05, Fig. 1 and **Table 2**).

**TABLE 2.** Changes in cardiovascular variables induced by different RTKI

RTKI	Dose (mg/kg)	Change in cardiovascular variable (h)					Target RTK and *K*_*d*_ (nM)^*a*^				
		HR	MAP	RVC	MVC	HVC	VEGFR2	RET	PDGFR-A	EGFR	KIT
Cediranib	Low: 3, i.v.	NE	+24	NE	−24	−24	1.1	6.1	0.4	230	
	High: 6, i.v.	NE	+24	−24	−2	−3					
Sorafenib	Low: 10, i.v.	NE	+3	NE	NE	−4	59	13	62	35	28
	High: 20, i.v.	+48	+24	NE	NE	−1
Vandetanib	Low: 12.5, i.p.	NE	+24	NE	NE	−26	820	34	230	9.5	
	High: 25, i.p.	NE	+24	NE	−48	−24					
Pazopanib	Low: 30, i.p.	NE	+24	−26	−24	NE	14	310	4.9		2.8
	High: 100, i.p.	NE	**+**24	NE	−24	−4				

**Figure 2.**
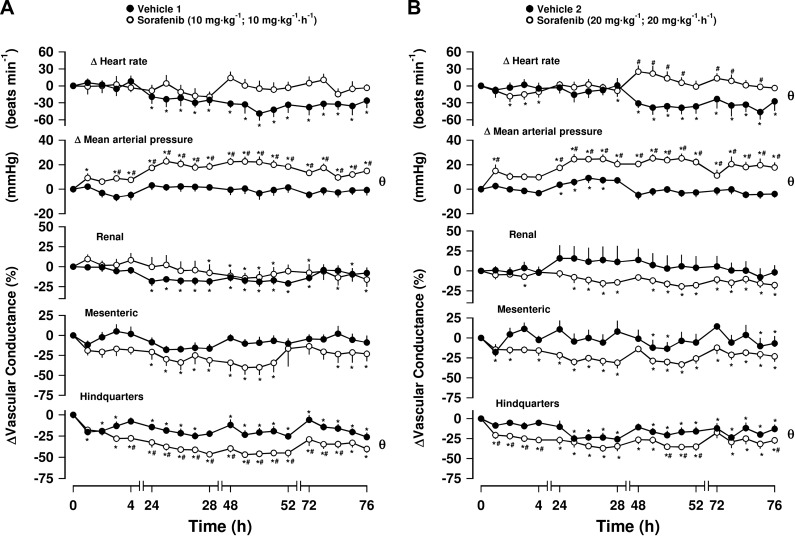
Cardiovascular responses to sorafenib in conscious, freely moving rats. Rats were dosed with either 10 mg/kg, i.v. (initial bolus followed by 10 mg/kg/h, i.v. infusion for 1 h; *n* = 8) (*A*) or 20 mg/kg, i.v. (initial bolus followed by 20 mg/kg/h, i.v. infusion for 1 h; *n* = 7) sorafenib (*B*). Vehicle controls (*n* = 7, *A*; *n* = 8, *B*) were administered 5% propylene glycol, 2% Tween 80 in sterile saline as described in Materials and Methods. These vehicle controls are same as those in corresponding Fig. 1*A, B*. Data points are means; vertical bars represent sem. *P < 0.05 *vs.* baseline (Friedman’s test; θ, significant between-group comparison, *P* < 0.05; Mann-Whitney *U* test, 0–76 h) based on integrated area under or above curve analysis. Where both above tests were positive, Mann-Whitney *U* test was conducted between treated and vehicle control groups at each timepoint. ^#^*P* < 0.05.

**Figure 3.**
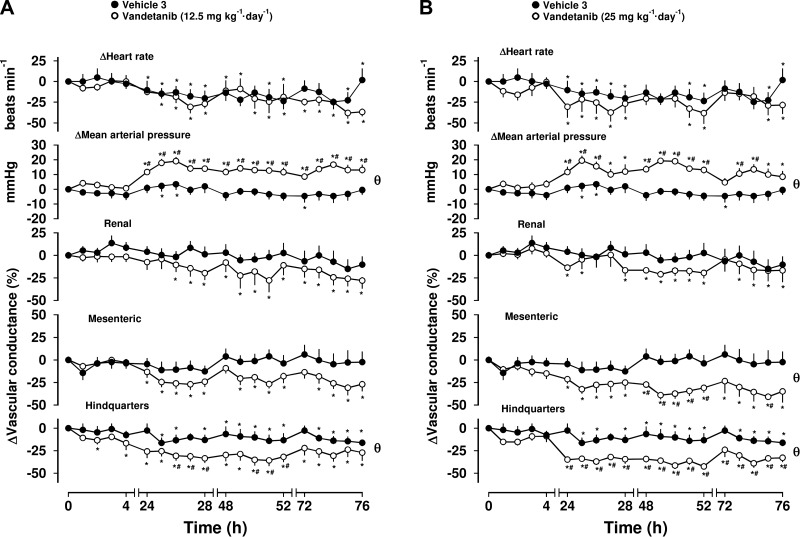
Cardiovascular responses to vandetanib in conscious, freely moving rats. Rats were dosed with either 12.5 mg/kg i.p. (*n* = 8) (*A*) or 25 mg/kg, i.p. (*n* = 8) vandetanib (*B*). Vehicle controls (*n* = 8) were administered 5% propylene glycol, 2% Tween 80 in sterile saline intraperitoneally, as described in Materials and Methods. These vehicle controls are identical in *A* and *B*. Data points are means; vertical bars represent sem. *P < 0.05 *vs.* baseline (Friedman’s test). θ, significant between-group comparison (*P* < 0.05; Mann-Whitney *U* test, 0–76 h) based on integrated area under or above curve analysis. Where both above tests were positive, Mann-Whitney *U* test was conducted between treated and vehicle control groups at each timepoint. ^#^*P* < 0.05.

**Figure 4.**
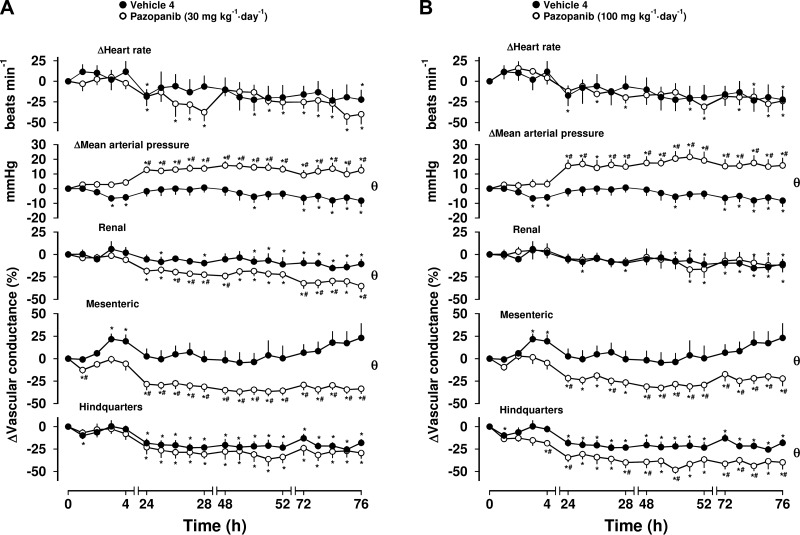
Cardiovascular responses to pazopanib in conscious, freely moving rats. Rats were dosed with either 30 mg/kg, i.p. (*n* = 8) (*A*) or 100 mg/kg, i.p. (*n* = 8) pazopanib (*B*). Vehicle controls (*n* = 8) were administered 5% propylene glycol, 2% Tween 80 in sterile saline intraperitoneally as described in Materials and Methods. These vehicle controls are identical in *A* and *B*. Data points are means; vertical bars represent sem. *P < 0.05 *vs.* baseline (Friedman’s test). θ, significant between-group comparison (*P* < 0.05; Mann-Whitney *U* test, 0–76 h) based on area under or above curve analysis. Where both above tests were positive, Mann-Whitney *U* test was conducted between treated and vehicle control groups at each timepoint. ^#^*P* < 0.05.

Significant changes in cardiovascular variables (*P* < 0.05, Mann-Whitney *U* test) indicated by between-group comparison (*P* < 0.05; Mann-Whitney *U* test) as indicated by θ in Figs. 1–4. Time (h) to earliest significant change in this experimental series (*P* < 0.05; Mann-Whitney *U* test) from corresponding time-matched vehicle control is also shown in parentheses (when it is also accompanied by significant change from basal levels). *^a^In vitro* *K_d_* values (nM) (31) reported on isolated proteins for VEGFR2 and other receptor tyrosine kinases. NE, no effect detected.

Sorafenib at 10 or 20 mg/kg also produced sustained increases in MAP relative to baseline values (**Fig. 2**, Friedman’s test). This pressor effect was accompanied by significant decreases in HVC, which were observed at both doses of sorafenib, although the changes at the higher dose of sorafenib were less consistent (Fig. 2, Friedman’s test). There was some mesenteric vasoconstriction (relative to basal; Freidman’s test), particularly after the higher dose of sorafenib, but the integrated change was not significantly different from vehicle, and there were no consistent significant changes (with respect to vehicle controls) in RVC (Mann-Whitney *U* test, integrated area under curve, 0–76 h; Fig. 2). No significant changes in HR were seen at the lowest dose of sorafenib, but a consistent and significant tachycardia (relative to vehicle controls) was observed at the highest dose after 48 h (Mann-Whitney *U* test, integrated area under curve, 0–76 h, Fig. 2*B*). The timecourse of the responses to sorafenib are summarized in Table 2.

### Series 2: Effects of vandetanib and pazopanib

Administration of vehicle in the control group for vandetanib caused no consistent cardiovascular effects, although there was some reduction in HR and HVC across the 4-d recording period (**Fig. 3**, Friedman’s test). Vandetanib (12.5 and 25 mg/kg, i.p.) produced a sustained increase in MAP (Fig. 3, Friedman’s test), which at the lower dose was accompanied by a significant fall in HVC only, whereas at the higher dose there was also a significant fall in MVC (Fig. 3, Friedman’s test). Comparison of integrated responses over the 76-h experimental period showed that these effects were significantly different from the vehicle group (Mann-Whitney *U* test, integrated area under curve, 0–76 h; Fig. 3). Differences from vehicle were observed between 24 and 26 h after the initiation of treatment (Mann-Whitney *U* test; Table 2). There were no consistent changes in RVC after either dose of vandetanib (Fig. 3, Friedman’s test). Moreover, neither dose of vandetanib caused a consistent change in HR.

Administration of vehicle in the control group for pazopanib did not evoke any consistent cardiovascular changes, except in the hindquarters vascular bed, where some vasoconstriction over the 4-d experimental period was apparent (**Fig. 4**, Friedman’s test). Pazopanib at 10 mg/kg, i.p. produced no consistent changes in MAP (*n* = 8; data not shown). However, at both 30 mg/kg, i.p. and 100 mg/kg, i.p., pazopanib evoked sustained increases in MAP, associated with pronounced mesenteric vasoconstrictions (Fig. 4, Friedman’s test). At the lower dose, a small but significant reduction in RVC was observed (Fig. 4*A*, Friedman’s test), which was not seen at the higher dose, although that dose did cause a hindquarters vasoconstriction (Fig. 4*B*, Friedman’s test). There were no consistent effects of pazopanib on HR at either dose (Fig. 4). Between-group comparison showed that the pressor responses to pazopanib at both doses were significantly different from vehicle over the 76-h experimental period. At the lower dose, the reductions in RVC and MVC were different from vehicle, whereas at the higher dose, the pazopanib-induced mesenteric and hindquarters vasoconstrictions were different from vehicle (Mann-Whitney *U* test, integrated area under curve, 0–76 h; Fig. 4). As with the other compounds, this was generally seen at and after 24 h (Mann-Whitney *U* test; Table 2).

### Series 3: Effects of losartan on the hypertension induced by cediranib

**Figure 5.**
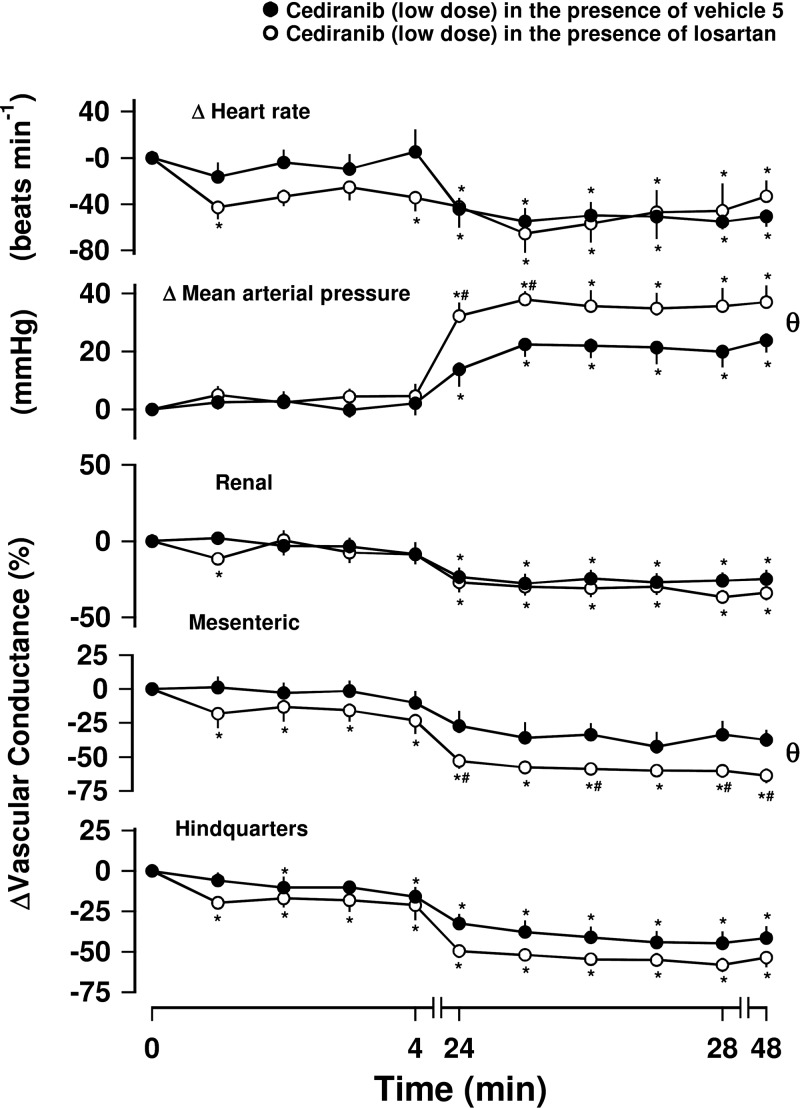
Effect of AT_1_R antagonist losartan on hypertension induced by cediranib. Rats were dosed intravenously with either losartan (10 mg/kg initial bolus, followed by 10 mg/kg/h continuous infusion; *n* = 8) or vehicle control (sterile saline; *n* = 6) over 5 h. After 1 h, each group was administered cediranib (3 mg/kg; 3 mg/kg/h infusion, i.v.). Data points are means; vertical bars represent sem. *P < 0.05 *vs.* baseline (Friedman’s test). θ, significant between-group comparison (*P* < 0.05; Mann-Whitney *U* test, 0–48 h) based on integrated area under or above curve analysis. Where both above tests were positive, Mann-Whitney *U* test was conducted between treated and vehicle control groups at each timepoint. ^#^*P* < 0.05.

In an attempt to unravel the mechanisms underlying the hypertension induced by the most potent RTKI, cediranib, we evaluated the effects of pretreatment with the AT_1_R antagonist losartan (28) on the cardiovascular responses to cediranib. In this series of experiments, cediranib produced similar effects on MAP and regional VC (relative to predrug baseline values; Friedman’s test) to those seen in earlier experiments (**Fig. 5**). The hypertensive and vasoconstrictor effects of cediranib were not inhibited by treatment with losartan; indeed, the effects on MAP and MVC were slightly enhanced (Mann-Whitney *U* test; Fig. 5).

### Series 4: Effects of endothelin-1 receptor antagonism or α- and β-adrenoceptor antagonism on responses to cediranib

The effects of antagonism of endothelin-1 receptor (24) or α- and β-adrenoceptors (29) on cediranib-induced pressor and regional vasoconstrictor effects were finally evaluated. Treatment with bosentan had no significant effects on the hemodynamic responses to cediranib compared to the vehicle group.

At the 48-h timepoint, animals treated with vehicle and low-dose cediranib showed a reduction in HR of 32 ± 9 beats/min, an increase in MAP of 30 ± 4 mmHg, and reductions in VC in the renal, mesenteric, and hindquarters vascular beds of −39.8 ± 6.9, −44.8 ± 5.0, and −58.0 ± 4.2%, respectively (*n* = 6). In the presence of bosentan, cediranib evoked a rise in MAP (29 ± 4 mmHg) and reductions in RVC, MVC, and HVC (−50.0 ± 5.3, −49.8 ± 3.8, and −56.3 ± 4.9%, respectively; *n* = 4), and these changes were not different from those seen in the vehicle and low-dose cediranib groups. The effect on HR was somewhat more variable over the experimental time period in the presence of bosentan, and at 48 h, HR was −12 ± 13 beats/min compared to baseline (immediately before the start of any treatment on d 1).

In the presence of bosentan, cediranib evoked a rise in MAP (29 ± 4 mmHg) and reductions in RVC, MVC, and HVC (−50.0 ± 5.3, −49.8 ± 3.8, and −56.3 ± 4.9%, respectively; *n* = 4). These changes were not different from those seen in the vehicle and low-dose cediranib group. The effect on HR was somewhat more variable over the experimental time period in the presence of bosentan, and at 48 h, HR was −12 ± 13 beats/min compared to baseline (immediately before the start of any treatment on d 1).

In the presence of phentolamine and propranolol, cediranib caused an elevation in MAP (34 ± 4 mmHg), which was accompanied by reductions in RVC, MVC, and HVC (−30.7 ± 12.9, −62.0 ± 2.9, and −54.8 ± 2.7%, respectively; *n* = 7). At the 48-h timepoint, there was a variable change in HR (−27 ± 21 beats/min). Compared to the vehicle and low-dose cediranib group, exposure to adrenergic blockade caused an enhanced cediranib-induced vasoconstrictor response in the mesenteric vascular bed (Mann-Whitney *U* test).

## DISCUSSION

In the present study, we, like others (23), have shown that in conscious rats, hypertension can be induced by RTKIs, as described in humans (13–17). Furthermore, we have been able to demonstrate for the first time that the onset of the hypertension is associated with regionally selective vasoconstrictions. The elevation in MAP was most consistently associated with a hindquarters vasoconstriction, although the mesenteric vascular bed was also affected. In all cases, RVC was least affected. Hypertension was observed with 4 different RTKIs that differ markedly in their ability to bind to VEGFR2 and inhibit functional activity (Table 2) (27, 31). For example, the most potent inhibitor of VEGFR2 used in the present study, cediranib (Table 2) (27, 31), produced an increase (within 24 h) in MAP. This was accompanied by consistent vasoconstrictions in both the hindquarters and mesenteric vascular beds (Table 2), with a decrease in RVC only apparent at the higher dose used (Table 2).

The increase in MAP was normally apparent within 24 h for all 4 RTKIs studied. This is rather different from the slower development of hypertension produced by antibody-based VEGF inhibitors in humans, particularly the monoclonal antibody bevacizumab, where the median interval from first dose of treatment to onset of hypertension ranged from 7–316 d (32). The time course of the development of hypertension with bevacizumab suggests that the mechanism of action may involve persistent changes in gene expression such as suppression of NO synthesis (15, 17, 33) and/or vascular rarefaction as a result of impaired angiogenesis over a longer period of time (15, 34–36). A role for NO in the ability of endogenous VEGF to maintain a low BP has been also suggested by previous work that showed that pretreatment with N-nitro-l-arginine methyl ester (l-NAME), an inhibitor of NO synthase, increased BP in control-treated mice to the same extent as that observed in animals treated with an anti-VEGFR2 (37). This observation, coupled with the finding that VEGFR2 treatment decreased eNOS and iNOS mRNA expression, suggests that VEGF may act to reduce BP *via* induction of NO and that VEGF inhibitor-mediated hypertension may be at least in part due to inhibition of NO (37).

Rarefaction is a reduction in the density of microvessels that leads to a decrease in vascular surface area and an increase in vascular resistance (15). However, it has been difficult to determine whether rarefaction is the cause or an effect of hypertension (35, 36).

Consistent with the findings presented here, more recent experimental and clinical studies have demonstrated RTKI-induced hypertensive effects within hours to days of treatment (38–41). The significant changes in MAP seen in the present study after 24 h with all 4 RTKIs are consistent with previous suggestions that the pressor effects may be due to changes in gene expression (15, 17, 33, 37), although the time course is probably too short for an involvement of vascular rarefaction (15, 34–36). There were variable effects of the different RTKIs on HR. We suggest that these inconsistent hemodynamic effects are likely to be due to their other kinase inhibitor properties, which are different for the 4 RTKIs studied (Table 2).

The most striking and original finding in the current study, however, was the hindquarters vasoconstriction that was observed over the same time course as the change in BP with all 4 RTKIs. As such, this is likely to be a major contributor to the observed increase in MAP with these drugs. There was also a tendency for these effects on HVC to occur earlier at the higher doses of RTKIs (Table 2). More variable effects were seen in MVC, with significant differences between the RTKIs studied. Thus, consistent vasoconstrictions in the mesenteric vascular bed were seen with cediranib and pazopanib, but less so with sorafenib and vandetanib. These differences are likely to be due to their different affinities for a range of other tyrosine kinases (27), some of which are illustrated in Table 2. The effects of all RTKIs were less consistent in the renal vascular bed, although at high doses there was some evidence of a vasoconstrictor effect, particularly with the more potent RTKIs, such as cediranib. This may point toward an autoregulatory role within the kidney (42) that goes some way toward compensating for the RTKI-induced reduction in RVC.

Interestingly, the results described herein are similar to previous reports of regional hemodynamic changes in rats after chronic NO synthase inhibition with l-NAME (43), where it was shown that l-NAME infusion caused an elevation in MAP, together with significant vasoconstriction in the hindquarters vascular bed. There was also a reduction in MVC, but little effect on RVC (43). This would be consistent with a role for NO in the mechanism by which endogenous VEGF is able to contribute to the homeostatic control of MAP *via* activation of VEGFR2 and subsequent calcium mobilization in vascular endothelial cells. The *in vivo* regional hemodynamics model developed here with RTKIs will allow future mechanistic studies to be undertaken to explore this in more detail.

Previous observations have suggested that the hypertension that develops in humans with bevacizumab can be managed with standard antihypertensive drugs (32). For example, there are reports that combinations of sympathetic blockade, in addition to diuretics, and renin angiotensin system inhibitors may be safe and efficacious for use in lowering BP in patients treated with anti-VEGF cancer therapies (44). In order to investigate whether the hypertension and other cardiovascular effects associated with RTKIs could also be prevented by antagonism of the actions of angiotensin II, endothelin-1, or catecholamines (21), we here investigated the impact of drugs that interfere with the actions of these signaling pathways.

Losartan treatment did not attenuate the increase in BP induced by cediranib. Indeed, there was greater elevation in MAP induced by cediranib in the presence of this AT_1_R antagonist. This pressor effect was accompanied by an increase in the mesenteric vasoconstriction normally observed with cediranib, although it is likely that losartan itself reduced BP and caused a slight mesenteric vasodilatation, thereby contributing to an enhanced change. These effects were apparent after 24 h. The lack of antagonism by losartan is consistent with other studies that demonstrated that inhibition of angiotensin II signaling with an angiotensin-converting enzyme inhibitor, captopril, was not able to offset the BP-elevating effects of sunitinib in rats instrumented with radiotelemetric devices (45). However, it should be acknowledged that other studies have shown that when the BP effects of an RTKI are marginal (that is, an elevation of ≤10 mmHg), a high dose of captopril was reported to completely reverse the rise in BP in a rat model (46).

Previous work with the endothelin-1 receptor antagonist macitentan has suggested that it can attenuate the increase in BP induced by sunitinib (45). However, in the present study, administration of the endothelin-1 receptor antagonist bosentan (24) did not produce any significant attenuation of the increase in MAP induced by cediranib. Similarly, antagonism of both α- and β-adrenoceptors with a combination of the nonselective α-antagonist phentolamine and the β-blocker propranolol (29) also had no effect on the increase in MAP induced by cediranib. There was, however, a noticeable enhancement of the mesenteric vasoconstrictor actions of cediranib in the presence of phentolamine and propranolol. The observation that the reduction in MVC is enhanced during adrenergic receptor blockade is compatible with the idea that the rise in BP is associated with a decrease in sympathetic tone counteracting the rise in BP.

It is clear from the foregoing discussion that interference with the actions of endothelin-1, angiotensin II, or catecholamines did not prevent the hypertension or the regionally selective vasoconstrictions induced by cediranib. There is therefore no evidence from our nonclinical animal model to suggest that antihypertensive drugs aimed at the sympathetic nervous system or hormones acting on endothelial cells will be effective in the treatment of hypertension induced by RTKIs. These data point to an urgent need to understand the mechanisms underlying the specific effects of RTKIs on regional blood flow, in particular in vascular beds that lead to a generalized increase in MAP, and the need to consider new antihypertensive strategies for patients being treated with these agents. The regional hemodynamic effects of RTKIs observed in conscious, freely moving rats in the present study suggest that this *in vivo* translational model provides an exquisitely sensitive and powerful approach with which to systematically unravel the complexities involved.

## Abbreviations

AT_1_R: angiotensin II type 1 receptor
BP: blood pressure
HR: heart rate
HVC: hindquarters vascular conductance
l-NAME: N-nitro-l-arginine methyl ester
MAP: mean arterial pressure
MVC: mesenteric vascular conductance
RTKI: receptor tyrosine kinase inhibitor
RVC: renal vascular conductance
VC: vascular conductance
VEGFR2: VEGF receptor 2
